# The importance of coastal gorgonians in the blue carbon budget

**DOI:** 10.1038/s41598-019-49797-4

**Published:** 2019-09-19

**Authors:** Martina Coppari, Chiara Zanella, Sergio Rossi

**Affiliations:** 1 Department of Earth, Environment and Life Sciences (DISTAV), University of Genoa, Genoa, Italy; 2 Institute of Environmental Science and Technology (ICTA), Autonomous University of Barcelona, Cerdanyola del Vallès, Spain; 3 Institute of Marine Sciences (ICM), Barcelona, Spain; 4 Department of Biological and Environmental Sciences and Technologies (DiSTeBA), University of Salento, Lecce, Italy

**Keywords:** Community ecology, Ecosystem ecology

## Abstract

Terrestrial (trees, shrubs) and marine (seaweeds and seagrasses) organisms act as carbon (C) sinks, but the role of benthic suspension feeders in this regard has been largely neglected so far. Gorgonians are one of the most conspicuous inhabitants of marine animal forests (mainly composed of sessile filter feeders); their seston capture rates influence benthic-pelagic coupling processes and they act as C sinks immobilizing carbon in their long-living structures. Three gorgonian species (*Paramuricea clavata*, *Eunicella singularis* and *Leptogorgia sarmentosa*) were studied coupling data of population size structure, biomass and spatial distribution in a NW Mediterranean area (Cap de Creus, Spain) with feeding, respiration and growth rates. In the study area, we calculated that *P. clavata* sequestered 0.73 ± 0.71 g C m^−2^ year^−1^, *E. singularis* 0.73 ± 0.89 g C m^−2^ year^−1^ and *L. sarmentosa* 0.03 ± 0.02 g C m^−2^ year^−1^. To our knowledge, this is the first attempt to calculate the importance as C sinks of gorgonian species that we consider as a starting point to estimate the importance of marine animal forests in C sequestration, and to ensure appropriate management and protection especially in areas and at depths where they are concentrated.

## Introduction

There is a large quantity of literature on the importance of terrestrial ecosystems as carbon (C) sinks^1^: the quantity of C removed by trees^2^, bushes and other terrestrial vegetation on land, and even by the soil^3,4^ has been already estimated. We can also calculate the proportion of the anthropogenic carbon (from industry, transport and agriculture/farming) that is retained in long-living structures^5^. In marine environments, previous studies are limited and focus mainly on carbon removal by coastal ecosystems (i.e. blue carbon^6^) such as mangroves^7^, seagrass meadows^8,9^ and tropical shallow coral reefs^10^, while other benthic communities appear to have been somewhat neglected.

Despite the presence of marine animal forests^11,12^ in all the oceans, in shallow and especially deeper areas (where light is no longer the direct driver of productivity), we still know little concerning the importance of benthic suspension-feeding organisms in carbon removal from the water column. Previous studies^13^ have already affirmed the importance of animals as carbon sinks in the overall C equation, but our concern is the specific role of marine animal forests in carbon sequestration and whether these ecosystems are a key factor in the overall biogeochemical cycle of the planet. This lack of knowledge represents a huge deficit in our comprehension of the global carbon cycle, and specifically of the processes of carbon sequestration.

Gorgonians are one of the most conspicuous organisms in tropical, temperate and polar marine animal forests^14–16^, and they are considered to be “eco-engineers” due to their ability to modify the surrounding habitat^17^. Except for mixotrophic species, gorgonians are passive suspension feeders that rely completely on ambient flow and particle abundances in the water masses for feeding^18^. Their diet is mainly composed of zooplankton^19–21^, microplankton^22,23^ and detrital particulate organic matter (POM)^24,25^. Through their feeding, they may influence the characteristics of surrounding waters especially when they reach high abundances^26^. They may also sequester large quantities of C from the water column and accumulate it in structural molecules, both organic and inorganic^27^.

The role of gorgonian species as C sinks^28^ may last for decades or centuries due to the high longevity of these organisms^29,30^. However, few studies to date have quantified the amount of C ingested and sequestered^19^ and, to the best of our knowledge, no studies have sought to undertake these estimates.

As a case study, we selected a pre-coralligenous and a coralligenous community in a well-known NW Mediterranean coastal area (Cap de Creus, Spain) dominated by three of the most representative gorgonian species in the Mediterranean Sea: *Paramuricea clavata* (Risso, 1826), *Eunicella singularis* (Esper, 1794) and *Leptogorgia sarmentosa* (Esper, 1789). *Paramuricea clavata* is a non-symbiotic species that mostly inhabits vertical rocky bottoms dominated by strong currents^15,31^. This species can reach large sizes^19,32^, especially in deep areas^33^, playing a significant role in benthic-pelagic coupling processes^34^ (i.e. the linkages between benthic and pelagic environments in aquatic systems). The main component of the diet of *P. clavata*^19^ is zooplankton, together with other seston particles (both live and detrital)^22^. *Eunicella singularis* is the only symbiotic gorgonian species in the Mediterranean Sea. This octocoral has two different morphotypes depending on depth^35^: the shallow type, with a candlestick-like shape and a brownish-white colour due to the presence of symbiotic algae, is mixotrophic^20,36^. Deeper colonies (generally located below 35 m depth) vary in shape, are of a bright white colour due to the lack of symbionts^35,37^ and rely solely on heterotrophic feeding. *Leptogorgia sarmentosa* is a non-symbiotic species normally living on boulders settled on soft bottoms and gravel benthic habitats at depths between 15–200 m^38^. Its diet is mainly composed of zooplankton^21^ and nano- and microplankton as well as detrital POM^23^. The spatial distribution and abundance of these three species was coupled with C flux data (energy input and output) for the three species in order to obtain their impact in benthic-pelagic coupling processes and assess their role as C sinks in accordance with the methodology of Coppari *et al*.^28,39,40^. Reliable data on marine benthic species distribution and abundance over large geographical areas can be obtained by video analysis conducted using Remotely Operated Vehicles (ROV), which enable the study of large areas at great depths with no impact on benthic communities^41^. ROV samplings are the ideal tool to describe the health status of benthic communities both qualitatively and quantitatively^42,43^, allowing for the characterization of the distribution patterns and population size structure^35,39^ of various species. Data on C assimilation are obtained by means of *in situ* or laboratory feeding and respiration experiments^40^.

To estimate the impact of the three gorgonian species on benthic-pelagic coupling processes (i.e. the C flux generated by these species), we have selected the most productive season (spring), in order to obtain a snapshot of the moment with the highest quality and quantity of food reaching the sea floor^12^. In warm temperate seas, spring represents the moment when benthic suspension feeders have the greatest impact on the water column, accumulating matter and energy which is then used during the rest of the year^12,34,39^.

This study aims to calculate the role played by these three gorgonian species in benthic-pelagic coupling processes in spring and as potential C sinks (blue carbon). To achieve this, the study was organized as follows: (1) collection of all previous literature data on the distribution and feeding, respiration and growth rates of the three studied species; (2) calculation of the total biomass of the three species in the study area, based on distribution and abundance data; (3) estimation of the role that the three gorgonian species play in benthic-pelagic coupling processes and in the C sequestration.

## Results

### *Paramuricea clavata*

A total of 269 *P. clavata* colonies were observed in shallow waters (0–35 m depth), accounting for a total biomass of 1269.66 g ash free dry mass (AFDM) (19.16 ± 16.62 g AFDM m^−2^, mean ± standard deviation). The total ingestion of C derived from zooplankton and microplankton feeding was 10.04 g C d^−1^ (0.15 ± 0.13 g C m^−2^ d^−1^). The total C respired by the shallow *P. clavata* colonies was 5.14 g C d^−1^ (0.08 ± 0.07 g C m^−2^ d^−1^), the balance between ingested and respired C being 4.90 g C d^−1^ (0.07 ± 0.06 g C m^−2^ d^−1^), equivalent to 441.35 g C (6.66 ± 5.78 g C m^−2^) in spring.

A total of 366 *P. clavata* colonies were observed in deep waters, corresponding to 1258.64 g AFDM (13.41 ± 13.91 g AFDM m^−2^). The total C ingested (by feeding on zooplankton and microplankton) was 9.73 g C d^−1^ (0.10 ± 0.11 g C m^−2^ d^−1^). The amount of C respired by the deep colonies was 3.40 g C d^−1^ (0.04 ± 0.04 g C m^−2^ d^−1^), the balance between ingested and respired C for all the deep *P. clavata* colonies being 6.34 g C d^−1^ (0.07 ± 0.07 g C m^−2^ d^−1^), corresponding to 570.18 g C (6.08 ± 6.30 g C m^−2^) in spring. Together, the observed shallow and deep *P. clavata* colonies are responsible for a net C flux of 1.01 kg C (6.28 ± 6.10 g C m^−2^) in spring.

**Figure 1:**
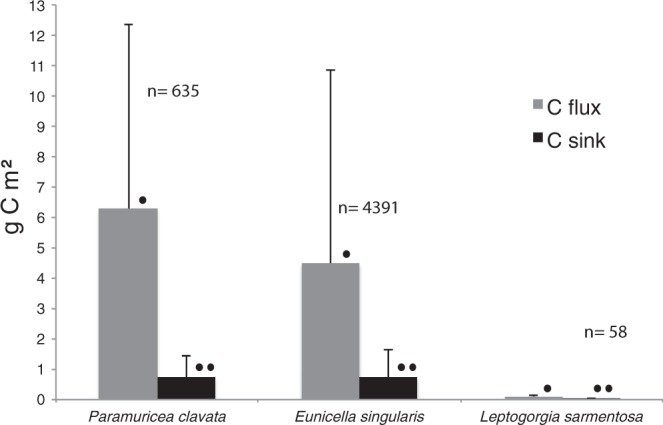
C flux (ingested-respired) and C sink (C invested in growth) by the three gorgonian species. • Indicates data expressed in g C m^2^ in spring, and •• indicates data expressed in g C m^2^ year^−1^; n = the number of colonies per species recorded in the 76 video transects.

The C sequestration attributed to growth by the observed *P. clavata* was 118.79 g C year^−1^ (0.73 ± 0.71 g C m^−2^ year^−1^), i.e. 57.42 g C year^−1^ (0.87 ± 0.75 g C m^−2^ year^−1^) and 61.36 g C year^−1^ (0.65 ± 0.68 g C m^−2^ year^−1^) for shallow and deep colonies respectively. These results are summarized in Fig. 1 and Table 1.

**Table 1:** Biomass, carbon flux (ingestion – respiration) and sink (growth) of the observed specimens of the three gorgonian species in the study area.

*Paramuricea clavata*	
Number of colonies*	635
Density (colonies m^−2^) ± SD	3.96 ± 3.84
Biomass (g AFDM)*	2528.30
Biomass m^−2^ (g AFDM m^−2^) ± SD	15.47 ± 15.07
Spring C flux (g C)*	1011.53
Spring C flux m^−2^ (g C m^−2^) ± SD	6.28 ± 6.09
Annual C sink (g C year^−1^)*	118.78
Annual C sink m^−2^ (g C m^−2^ year^−1^) ± SD	0.73 ± 0.71
***Eunicella singularis***	
Number of colonies*	4391
Density (colonies m^−2^) ± SD	5.75 ± 7.24
Biomass (g AFDM)*	2256
Biomass m^−2^ (g AFDM m^−2^) ± SD	2.90 ± 3.72
Spring C flux (g C)*	3516.23
Spring C flux m^−2^ (g C m^−2^) ± SD	4.48 ± 6.36
Annual C sink (g C year^−1^)*	559.14
Annual C sink m^−2^ (g C m^−2^ year^−1^) ± SD	0.73 ± 0.89
***Leptogorgia sarmentosa***	
Number of colonies*	58
Density (colonies m^−2^) ± SD	0.63 ± 0.30
Biomass (g AFDM)*	16.07
Biomass m^−2^ (g AFDM m^−2^) ± SD	0.17 ± 0.08
Spring C flux (g C)*	8.75
Spring C flux m^−2^ (g C m^−2^) ± SD	0.10 ± 0.04
Annual C sink (g C year^−1^)*	2.96
Annual C sink m^−2^ (g C m^−2^ year^−1^) ± SD	0.03 ± 0.02

*Refers to 1.14 ha covered by ROV.

### *Eunicella singularis*

In the 76 ROV transects, 2229 *E. singularis* colonies were recorded at shallow depths, representing a total biomass of 1629.15 g AFDM (2.95 ± 4.13 g AFDM m^−2^). The total amount of C ingested (autotrophic and heterotrophic feeding) was 36.40 g C d^−1^ (0.07 ± 0.09 g C m^−2^ d^−1^). The total C respired was as high as 4.28 g C d^−1^ (0.01 ± 0.01 g C m^−2^ d^−1^). The balance between ingested and respired C for the observed shallow *E. singularis* colonies of Cap de Creus was thus 32.11 g C d^−1^ (0.06 ± 0.08 g C m^−2^ d^−1^), equivalent to 2890.24 g C in spring (5.23 ± 7.33 g C m^−2^). We recorded 2162 deep *E. singularis* colonies with a total biomass of 627.37 g AFDM (2.79 ± 2.56 g AFDM m^−2^). The total amount of C ingested (heterotrophic feeding) was 7.80 g C d^−1^ (0.03 ± 0.03 g C m^−2^ d^−1^). The total C respired by the observed *E. singularis* was 0.85 g C d^−1^ (0.004 ± 0.003 g C m^−2^ d^−1^). The balance between ingested and respired C for the observed deep colonies of *E. singularis* was thus 6.96 g C d^−1^ (0.03 ± 0.03 g C m^−2^ d^−1^), equivalent to 625.99 g C (2.79 ± 2.55 g C m^−2^) in spring. The observed shallow and deep colonies are responsible for a total net C flux of 3.52 kg C (4.48 ± 6.36 g C m^−2^) in spring. They sequestered 559.37 g C year^−1^ (0.73 ± 0.89 g C m^−2^ year^−1^): 322.77 (0.58 ± 0.82 g C m^−2^ year^−1^) and 236.6 g C (1.05 ± 0.96 g C m^−2^ year^−1^) invested in growth by shallow and deep colonies, respectively. These results are summarized in Fig. 1 and Table 1.

### *Leptogorgia sarmentosa*

23 colonies of *L. sarmentosa* were observed in shallow waters, accounting for a total biomass of 6.46 g AFDM (0.19 ± 0.10 g AFDM m^−2^). The total ingestion of C derived from zooplankton and microplankton feeding was 0.06 g C d^−1^ (0.002 ± 0.001 g C m^−2^ d^−1^). The total C respired by the shallow *L. sarmentosa* colonies was 0.02 g C d^−1^ (0.001 ± 0.0004 g C m^−2^ d^−1^), and the balance between ingested and respired C was thus 0.04 g C d^−1^ (0.001 ± 0.001 g C m^−2^ d^−1^), equivalent to 3.17 g C in spring (0.09 ± 0.05 g C m^−2^). 35 colonies of *L. sarmentosa* were observed in deep waters, which corresponded to 9.61 g AFDM (0.17 ± 0.07 g AFDM m^−2^). The total amount of C ingested by feeding on zooplankton and microplankton was 0.09 g C d^−1^ (0.002 ± 0.001 g C m^−2^ d^−1^). The amount of C respired by the deep colonies was 0.03 g C d^−1^ (0.0004 ± 0.0002 g C m^−2^ d^−1^), and the balance between ingested and respired C for the deep colonies of *L. sarmentosa* was thus 0.06 g C d^−1^ (0.001 ± 0.0005 g C m^−2^ d^−1^), corresponding to 5.58 g C in spring (0.10 ± 0.04 g C m^−2^). Together, the observed shallow and deep colonies of *L. sarmentosa* are responsible for a total net C flux of 8.75 g C in spring (0.10 ± 0.04 g C m^−2^).

The C invested in growth by the observed *L. sarmentosa* was 2.96 g C year^−1^ (0.03 ± 0.02 g C m^−2^ year^−1^), i.e. 1.18 (0.03 ± 0.02 g C m^−2^ year^−1^) and 1.78 g C year^−1^ (0.03 ± 0.01 g C m^−2^ year^−1^) for shallow and deep colonies, respectively. These results are summarized in Fig. 1 and Table 1.

### Potential role as C sinks of the three gorgonian species

The potential biomass, C flux and C sequestered are listed in Supplementary Data S1a. *P. clavata* sequestered and immobilized as living tissue a quantity of C as high as 2.58 kg C ha^−1^ year^−1^, whereas *E. singularis* sequestered 8.90 kg C ha^−1^ year^−1^ and *L. sarmentosa* 0.02 kg C ha^−1^ year^−1^.

### Statistical analysis

ANOVA highlighted a significant difference of the biomass of the three species (F = 128.1; p-value < 0.01), and Tukey test showed that, in terms of biomass, every species differed from the other (*L. sarmentosa* × *E. singularis*, p-value < 0.01; *P. clavata* × *E. singularis*, p-value < 0.01; *P. clavata* × *L. sarmentosa*, p-value < 0.01).

The C flux mediated by the three gorgonians was significantly different among the species (ANOVA: F = 20.02; p-value < 0.01; Tukey test: *L. sarmentosa* × *E. singularis*, p-value < 0.01; *P. clavata* × *E. singularis*, p-value < 0.01; *P. clavata* × *L. sarmentosa*, p-value < 0.01).

The amount of C sequestered by the three species was significantly different (ANOVA: F = 20.02; p-value < 0.01) with Tukey test highlighting differences in the C sequestration among *L. sarmentosa* and *E. singularis* and among *P. clavata* and *L. sarmentosa* (p-value < 0.01), whilst no significant difference was observed among *P. clavata* and *E. singularis* (p-value 0.714) (Fig. 1).

## Discussion

The present study aimed to quantify the importance of the organisms constituting marine animal forests as contributors to blue carbon storage. The methodology presented here combines data from laboratory and *in situ* feeding experiments and data about marine species presence, density and population structure quantified with ROV methods. This is the first time that such an approach has been used with key species of a marine animal forest and could be applied and replicated in other benthic ecosystems as long as data on species biology and distribution are available.

*Eunicella singularis* was the most abundant gorgonian species in the study area (1.14 ha). However, despite having the highest number of observed colonies (4391 *E. singularis* vs. 635 *Paramuricea clavata* in 1.14 ha), this species had slightly less (although significative) biomass than *P. clavata* (2256.52 g vs. 2528.30 g). Indeed, *P. clavata* had a density of organic material (AFDM cm^−1^) three times higher than *E. singularis*. Even though *P. clavata* is restricted to certain substrates (i.e. rocky bottoms) at certain inclinations^15^, this species reaches greater sizes and gathers more biomass than the more abundant *E. singularis*. *Leptogorgia sarmentosa* accounted for the smallest number of observed colonies (58) and the smallest biomass (16.07 g AFDM in the entire study area). This low abundance is probably due to the consequences of anthropogenic activities in soft and gravelly bottoms, being the density much higher in marine protected areas^44^. Indeed, fishing practices (e.g. gill nets and trawling) represent a threat to gorgonians, directly reducing their abundance (gorgonians are usually caught as by-catch or by direct harvesting) or indirectly influencing their reproductive output^45,46^, which in turn reduces their contribution to benthic-pelagic coupling processes and C acquisition.

The autotrophic contribution of symbiotic algae in *E. singularis*^36^, together with its higher abundance, make this species more important in benthic-pelagic coupling processes than the other two heterotrophic species in spring (Table 1). Indeed, in the whole study area *E. singularis* is responsible for a C flux of 3.52 kg C whilst *P. clavata* and *L. sarmentosa* account for 1.01 and 0.01 kg C respectively. The recent study by Ferrier-Pagés *et al*.^36^ highlights the importance of autotrophic C supply for this mixotrophic gorgonian, which, in shallow areas, has low C input from zooplankton and other prey^20^. The mixotrophic habits of *E. singularis* might explain its wider distribution, limited only by the need for suitable hard substrata^47^, whereas *P. clavata*, which relies on zooplankton and detritus as its main food source^19^, needs hard bottoms exposed to strong currents that ensure the provision of food^15,32,38^. It should be pointed out that not all the C assimilated is subsequently released into the water column via respiration. Indeed it is also used for reproduction and growth and thus fixed as biomass. The ingested C used for reproduction will be released into the water column as gametes or larvae^34^ which might be preyed on before fertilization or settlement, influencing benthic-pelagic coupling processes^48^. Another possibility for these larvae is to find a suitable substrate, undergoing metamorphosis and becoming a new C sink.

Spring is the most favourable season for Mediterranean benthic suspension feeders due to the high quality and quantity of the near-bottom seston^49^, which enable increased activity^50^ and secondary production^51^, as well as the accumulation of large quantities of lipids essential for surviving less favourable seasons^52,53^. Spring represents the only period of the year in which water column productivity allows for energy storage, whereas during the other seasons of the year, energy outputs exceed energy inputs^39^. It is reasonable to think that this is a feature of many active and passive suspension feeders^34,51^. Indeed, in other highly seasonal systems such as Antarctic benthos, metabolic activity is concentrated in late spring and summer^54^, with autumn representing the moment when benthic suspension feeders have the largest stores of energy from phytoplankton blooms^55^, although we cannot exclude the presence of short food pulses during the rest of the year^50^. Although these food pulses may be more important than previously thought in estimating the energy available for suspension feeders, they are rarely captured by monthly sampling^45^. The lack of an adequate method to evaluate these food pulses might have caused an underestimation of the role played by the three gorgonian species in C flux.

The minor importance of *L. sarmentosa* to benthic-pelagic coupling in spring in our study area (only 8.75 g C in 1.14 ha) is related to the lower abundance of this heterotrophic gorgonian. It has been shown that the impact of individual colonies on the near-bottom seston is not negligible^21^, and the only reason for the low impact of this octocoral in this area is thus the presence of few colonies. Indeed, this species only grows on gravel or small boulders resting on soft bottoms, and even on biogenic substrates^21,56^, which were often frequented by trawlers in the Cap de Creus area before the establishment of the MPA (Marine Protected Area)^57^. In zones were bottom trawling is not permitted or is avoided due to the presence of large rocky formations, the density may reach up to 8 colonies m^−2^ ^56^. This species may be very abundant in soft bottom gravel zones, from a depth of 30 to 150 meters^58^, and the resulting impact on C flux and sequestration might be much higher. The impact of bottom trawling and fishing activities on animal forests is one of the main concerns of conservation strategies^59–61^, with some species being especially threatened. Indeed, the change in the population structure of the rare octocoral *Corallium rubrum* due to overharvesting in the same area^62^ is claimed to influence the benthic-pelagic coupling processes and C sequestration mediated by this species (Mallo personal observation). Calculation of the impact on the water column of this octocoral in the Cap de Creus area demonstrates that this species is no longer the C sink that it would be in non-harvested populations^28^.

In terms of their value as C sinks, in the study area *P. clavata*, *E. singularis* and *L. sarmentosa* sequestered and fixed as biomass 118.78, 559.14 and 2.96 g C year^−1^, respectively. *L. sarmentosa* is the species with the highest C investment in growth (Fig. 1). This gorgonian is characterised by fast growth of primary and secondary branches, as well as high plasticity, resulting in the conversion of planktonic C to tissue and the loss of biomass (e.g. shedding of apical branches) in adverse conditions^63^. In contrast, both *P. clavata* and *E. singularis* show lower growth of primary branches, with limited subsequent loss of tissue^34,64^. Thus, the value as a C sink varies among species (and possibly within the same species among areas, but this variation is even more difficult to calculate).

To estimate the C invested in growth by *E. singularis*, data from studies performed in Banyuls-sur-Mer were used^64^, as well as observations from two Italian areas^65^ which are more conservative than the recent observations by Viladrich *et al*.^47^ in the Cap de Creus area. Consequently we possibly underestimated the role of C sink played by this species in the study area, at least during its early life stage. Differences in the C sequestration between species is to be considered when an overall estimate of Marine Animal Forests C sink is calculated; indeed, these ecosystems may be composed of very different suspension feeding organisms, representing hotspots of biodiversity all over the world^12^.

C sequestration by the three gorgonian species also varies with depth (Supplementary Data S2). Indeed, shallow *E. singularis* colonies accounted for the highest ingestion and consequently retention of C due to their abundance in the study area. The shallow and deep populations of this species have been shown to differ in terms of reproduction, energy storage and trophic ecology (adopting mixotrophy and heterotrophy in shallow and deep patches respectively^66^). For the other two gorgonians, both heterotrophic, the opposite trend was observed, with the deep colonies sequestering more C. These results are related to the higher number of colonies observed at greater depths.

Regarding the broad-scale estimation of gorgonian abundances in the study area (based on their mean density and the total abundance of suitable benthic assemblages), *E. singularis* had the highest number of colonies (Table 1, Supplementary Data S1a and S2), one or two orders of magnitude higher than the other two species. This high number of potential colonies, mainly related to the presence of vast areas of suitable assemblages along the coast in this part of the Mediterranean, means that this species has the greatest influence in terms of benthic-pelagic coupling processes and C sequestration. Interestingly, despite the low number of *L. sarmentosa* colonies observed in the video transects, the potential number of colonies of this species is higher than the number of *P. clavata* colonies. Indeed, *L. sarmentosa* has a larger quantity of suitable substrate than the other species (Supplementary Data S1b), but, as already pointed out, trawling has had a significant impact on its ability to act as a C sink: every damaged or removed gorgonian is one that is no longer sequestering C and immobilizing it in its skeleton or tissues.

The role of both land and marine ecosystems as C sinks has been estimated by previous studies. Grace *et al*.^67^ pointed out that undisturbed Amazonian forests retain 1.02 t C ha^−1^ year^−1^; Duarte *et al*.^8^ calculated the amount of C accumulated by seagrass meadows to be as high as 6.7 t C ha^−1^ year^−1^; and Eong^68^ found that mangrove forests retain 1.5 t C ha^−1^ year^−1^. Our study presents the first approximation of animal forest-wide fluxes. The three gorgonian species sequestered, by growth, 1.15 × 10^−2^ t C ha^−1^ year^−1^, thus two orders of magnitude lower than the above-mentioned ecosystems. It should be stressed that the estimate performed in this study is just the first step towards a more complete understanding of the importance of animal forests in the blue carbon budget: only three gorgonian species have been considered. The amount of carbon invested in reproduction and the carbon lost through predation should be calculated and included in this estimate. The contribution of other organisms, whose recruitment is favoured by the presence of gorgonians forests (e.g. sponges, bryozoans, scleractinians and especially coralline algae^69,70^), and that constitute pre-coralligenous and coralligenous communities should also be considered, in order to calculate more accurately the importance of these ecosystems as C sinks. Further studies will allow this first calculation to be refined evaluating also the C loss determined by the turnover (e.g. natural mortality) of the species, and by predation. Such data are unfortunately not available for the three gorgonians species, reason why we did not consider these two processes in our estimations. A previous study reported a mass mortality event related to the increase of seawater temperature down to 50 m depth^71^, which caused the death of up to 60–100% of the colonies of the three gorgonians in the Ligurian Sea (NW Mediterranean). As a consequence of such an event their role as C sinks would be lost as well. But this particular situation is not representative of natural turnover rates. Despite the existence of these gaps of knowledge, which imply a urgent need of extensive investigations to properly estimate the role of marine animal forests worldwide, we believe that our results represents a starting point for the evaluation of the importance of benthic suspension feeders as C sinks, and their contribution to the blue carbon. Further studies will eventually refine our calculations that represent a first rough estimate of the marine animal forests as carbon sinks. The role of marine organisms in the C sequestration is currently understood as crucial to the fight against climate change^72^. In the near future, climate change will transform the marine animal forests, shaping the seascape and probably diminishing the capability to store C of these three dimensional alive structures^73^. Despite this, the biomass accumulated in suspension feeders’ living structures has not yet been considered in the overall budget of the oceans’ C cycle. These complex structures are subject to many stressors, with their complexity diminished up to ten times faster than land forests^11,12^. Consequently, we highlight again the need to consider their role as C sinks in the framework of protection measures. As already pointed out by other authors^74^, the implication may be revising or extending the depth limits of protection measures, especially in those areas in which marine animal forests are concentrated.

## Methods

### Study area

**Figure 2:**
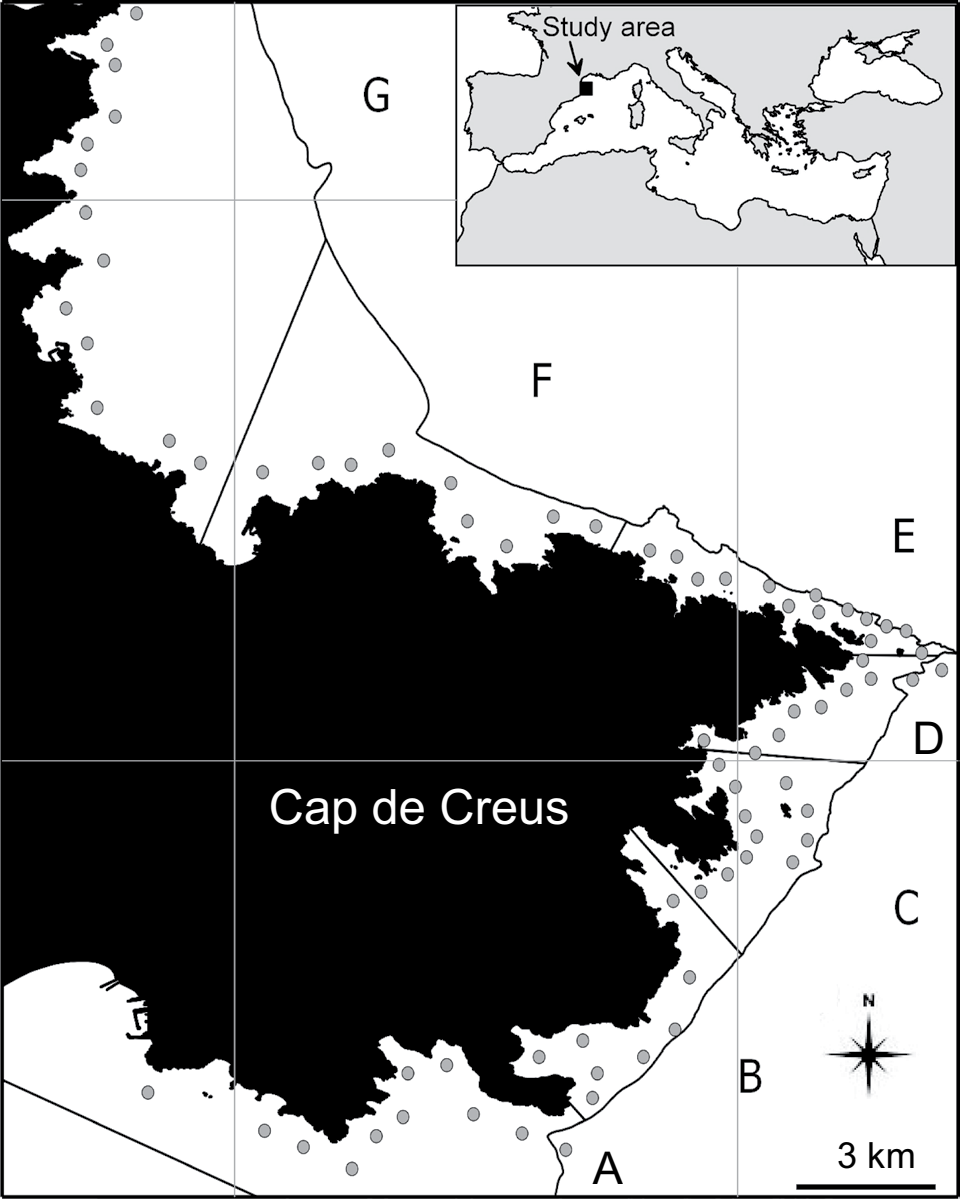
Map of the study area. Inset: location of the study area; Main Figure: Cap de Creus showing the seven subareas and transect positions (grey circles).

The study area is located in Cap de Creus (42°19′12″N; 3°19′34″E), in the north-western Mediterranean Sea (Fig. 2). A detailed description of the study area with the main hydrodynamic features is available in Gori *et al*.^15,35^. These authors analysed 76 video transects recorded by ROV and grouped them into seven subareas based on environmental and hydrodynamic conditions in the study area (Fig. 2). A detailed study of the benthic communities characterizing this area is available in Sardà *et al*.^44^, who also report the surface area (in ha) occupied by different benthic assemblage suitable for the studied species.

### Biometric relationships and C retention measurements

For each studied species, previous literature on spatial and bathymetrical distribution, population size structure and feeding, respiration and growth rates was reviewed (Supplementary Data S3). Literature data were expressed in a variety of units; hence we first applied transformations, based either on the already available biometric relationships for the three-studied species or on new ones, calculated in this study. The previously published and new biometric relationships, applied in this study, are summarized in Table 2.

**Table 2:** This table summarizes the biometric relationships used in the study.

		H-L	POL (mean ± SE)	AFDM (mean ± SE)	H-AREA
*Paramuricea clavata*		y = 1.06x^1.69^ ^78^	From 630 ± 139 to 26175 ± 5782^34^	18.35 ± 2.33^[*]^	
*Eunicella singularis*	S	y = 0.2869x^1.9652[*]^	31.1 ± 1.1^20^	5.69 ± 0.47^[*]^	y = 0.0609x^2.4655[*]^
	D	y = 0.4669x^1.8432[*]^		4.43 ± 0.37^[*]^	
*Leptogorgia sarmentosa*		y = 2.1167x^1.3684[*]^		2.94 ± 0.67^[*]^	

* Indicates that the relationship was calculated in this study. H = height of the colony,(cm); L = total length of the colony,(cm); POL = number of polyps; in^34^ number of polyps per colony’size class; in^20^ number of polyps per cm^−1^; AFDM = ash free dry mass (mg cm^−1^); AREA = total colony surface area (cm^2^). For *Eunicella singularis* the biometric relationships are calculated for both the shallow (S) colonies (thus considering the autotrophic contribution to the species’ feeding) and the deep (D) colonies. Empty spaces mean that the corresponding relationship was not used for that species.

**Figure 3:**
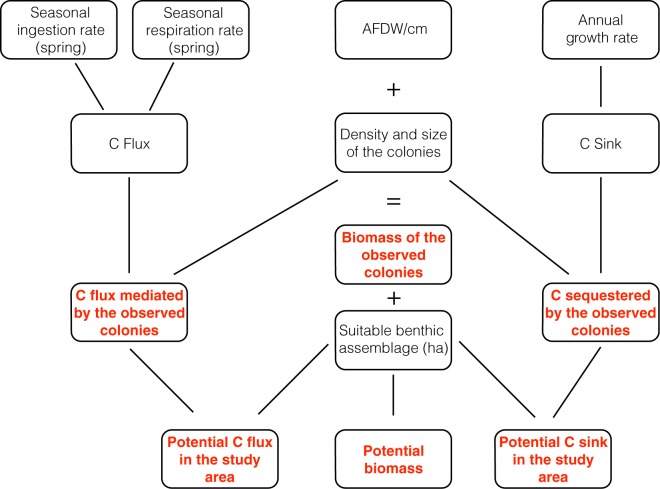
Flow chart summarizing the steps performed to calculate C flux and C sequestration as well as the total biomass of both the observed and estimated colonies in the study area.

For the three gorgonian species we followed the same procedures: (1) The observed colonies were divided into shallow (0–35 m) and deep (>35 m) by means of video analysis. This depth threshold was chosen with reference to the characteristics of the study area, in which light penetration at 35 m marks the shift from precoralligenous and shallow coralligenous communities to deep coralligenous communities^75,76^. (2) Where necessary, in order to compare past data on feeding, respiration and growth rates, measure unit transformations were performed. (3) C flux (difference among ingestion and respiration) and C sequestration of the observed gorgonians in 1.14 ha were obtained combining data on density on gorgonian patch (Table 3), biomass and population structure with data listed in point 2). (4) The potential number of colonies for the entire study area was estimated by quantifying the area (in hectares, ha) of suitable benthic assemblage per species in the Cap de Creus area^44^ (Supplementary Data S1a,b) and multiplying it by the mean total density of colonies (density on sampling units with and without gorgonians) (Table 3) in the investigated area, obtained from video transects. The most suitable conditions for each species were selected on the basis of previous studies of the ecology of the three gorgonians^31,37,75^. (5) C flux and sequestration of the estimated number of colonies in all the Cap de Creus area were obtained combining data listed in point (2) and (4). Figure 3 summarizes the steps performed in this study.

**Table 3:** Density (col m^−2^) ± standard deviation (SD) of the three gorgonian species per benthic assemblage.

Benthic assemblages	Total density ± SD (col m^−2^)	Density on patch ± SD (col m^−2^)
***Paramuricea clavata***		
Vertical coralligenous •	0.423 ± 1.562 n = 376	3.698 ± 3.063 n = 43
Platform coralligenous ••	1.000 ± 2.945 n = 118	4.370 ± 4.871 n = 27
***Eunicella singularis***		
Photophilic algal communities •	0.323 ± 1.840 n = 93	2.000 ± 4.318 n = 15
Precoralligenous •	3.129 ± 6.200 n = 167	5.559 ± 7.413 n = 94
Vertical coralligenous •	1.737 ± 4.109 n = 376	4.213 ± 5.533 n = 155
Platform coralligenous ••	6.631 ± 8.532 n = 118	9.543 ± 8.776 n = 82
***Leptogorgia sarmentosa***		
Photophilic algal communities •	0.005 ± 0.052 n = 93	0.500 n = 1
Precoralligenous •	0.021 ± 0.149 n = 167	0.875 ± 0.479 n = 4
Vertical coralligenous •	0.013 ± 0.109 n = 376	0.714 ± 0.393 n = 7
Platform coralligenous ••	0.017 ± 0.112 n = 118	0.667 ± 0.289 n = 3
Littoral sandy mud •	0.008 ± 0.063 n = 64	0.500 n = 1
Littoral medium and coarse sand •	0.013 ± 0.081 n = 113	0.500 ± 0.000 n = 3
Detrital littoral sands ••	0.015 ± 0.105 n = 423	0.591 ± 0.302 n = 11
Detrital littoral sandy mud ••	0.057 ± 0.193 n = 211	0.600 ± 0.262 n = 20

Second column shows the total density ± SD calculated in all the sampling units recorded from video analysis per benthic assemblage (with and without gorgonians); third column shows the density ± SD in the sampling units with the presence of the gorgonians (density on the patch). “n” indicates the number of sampling units analyzed per benthic assemblages (Gori unpublished). ^•^Indicates shallow benthic assemblages; ^••^Indicates deep benthic assemblages.

### *Paramuricea clavata*

Data on the spatial and bathymetric distribution of *P. clavata* in the study area are available in Gori *et al*.^15^. For shallow colonies, the population size structure found by Linares *et al*.^32^ was applied to the total number of shallow gorgonians observed in Gori *et al*.^15^. The size of deep colonies was measured and their population size structure assessed as in Gori *et al*.^35^.

The ingestion rates of detrital POM and pico-, nano-, and microplankton in *P. clavata* were studied in Ribes *et al*.^22^ and the contribution of zooplankton to the diet of this species was assessed in Coma *et al*.^19^. Respiration rates at 16 °C and 14 °C were used for shallow and deep colonies respectively^77^. For the three gorgonian species, oxygen consumption was converted into respired C using a conversion factor of 0.281^78^. Since the ingestion rates of *P. clavata* were expressed in μg C polyp^−1^ d^−1^ ^19,22^ and respiration rates in mg O_2_ g^−1^ AFDM h^−1^ ^77^, the relationship between colony height (cm) and total number of polyps, and the relationship between colony height and AFDM were calculated. Colony size was converted into number of polyps using the relationship obtained by Coma *et al*.^34^. Colony height (cm) was converted into colony linear length (hereafter length) (cm) following Coma^79^. To convert colony length (cm) into AFDM, 15 pieces of shallow *P. clavata* were randomly collected *in situ* in spring 2012 (Punta s’Oliguera, Cap de Creus, 42°17′1.62″N, 3°17′57.18″E) by SCUBA divers, at a depth of 15–20 m. Once in the laboratory, the tips of the branches were removed and the remaining portion was measured, dried at 90 °C for 24 h, weighed, and then combusted for 5 h at 450 °C and weighed again to determine its AFDM^52^. The AFDM was then normalized by the size of the portion collected. The AFDM measured for *P. clavata* was 18.35 ± 2.33 (mean ± standard error (SE)) mg cm^−1^, and this was used for calculating both the total biomass in the study area and the respiration.

Coma^79^ studied the growth rate per size class and the annual amount of C invested in growth by *P. clavata*. This value was used to estimate the total C invested in growth of this species, taking into account the number of colonies per size class in the study area (Supplementary Data S3).

### *Eunicella singularis*

Data on the spatial and bathymetric distribution of *Eunicella singularis* in the study area are available in Gori *et al*.^15^. The size of the shallow colonies was estimated from the population size structures found by Linares *et al*.^32^. For that of the deep colonies, the population size structure in subareas E and F had already been studied by Gori *et al*.^35^. The availability of the video transects analysed by Gori *et al*.^15^ allowed the study of the size structure of the deep populations located in the other subareas, following the same approach as in Gori *et al*.^35^.

Since shallow colonies of *E. singularis* host zooxanthellae, both autotrophic^36^ and heterotrophic^20^ feeding were considered to contribute to the total amount of C inputs. No previous data were available on the consumption by this species of detrital POC and pico-, nano- and microplankton. Therefore, this was estimated with reference to data on C ingestion by the gorgonian *Leptogorgia sarmentosa*^23^, considering that the two species share the same habitat and depth range. Similarly, since no information was available about the feeding rates of deep colonies of *E. singularis*, the heterotrophic feeding rates of shallow colonies^20,23^ were used, even though we were conscious of the introduction of some bias, i.e. the likely underestimation of deep heterotrophic feeding. Respiration rates at 16 °C for the shallow colonies were obtained from Previati *et al*.^77^. For the deep colonies, the respiration rates of the non-symbiotic species *E. cavolini* at 14 °C^76^ were applied. The ingestion rates of *E. singularis* were expressed in μg C polyp^−1^ h^−1^ and in μg C cm^−2^ d^−1^ for the zooplankton^20^ and the autotrophic contribution^36^ respectively. Data on microplankton ingestion^23^ and respiration rates^77^ were presented as μg C g AFDM^−1^ h^−1^ and mg O_2_ g AFDM^−1^ h^−1^ respectively. Consequently, the relationships between colony height (cm) and linear length of the colony (cm), colony height (cm) and total colony area (cm^2^), and colony length (cm) and AFDM per colony were calculated. For all these relationships (Supplementary Data S4), the 95% confidence limit was applied.

For shallow colonies, the first two relationships were obtained by means of image analysis (as in Coppari^28^): 28 colonies were photographed *in situ* next to a ruler, and the maximum height (i.e. the maximum distance between the base of the stem and the tips of the highest branches) and linear length of all the branches was measured with Macnification 1.8 software^80^. This relationship was found to be:

$${\rm{y}}=0.2869\,{{\rm{x}}}^{1.9652},\,{\rm{with}}\,{{\rm{R}}}^{2}=0.78,{p} \mbox{-} {\rm{value}} < 0.001,\,{\rm{n}}=28$$

where y = linear length of the colony and x = height of the colony (Supplementary Data S4a).

The total length of the colony was then converted into the number of polyps by means of the relationship obtained by Coma *et al*.^20^. By means of image analysis, the diameter and length of each branch were also measured, in order to calculate the surface area of the branches forming the colony, resulting in the following relationship between colony height (cm) and total colony surface area (cm^2^):

$${\rm{y}}=0.0609\,{{\rm{x}}}^{2.4655},\,{\rm{with}}\,{{\rm{R}}}^{2}=0.81,{p} \mbox{-} {\rm{value}} < 0.001,\,{\rm{n}}=28$$

where y = colony surface area and x = height of the colony (Supplementary Data S4b).

Still images of deep colonies of *E. singularis* were extracted from videos and measured following the same method explained above, obtaining the relationship between colony height (cm) and total colony length (cm) for deep colonies:

$${\rm{y}}=0.4669\,{{\rm{x}}}^{1.8432},\,{\rm{with}}\,{{\rm{R}}}^{2}=0.66,{p} \mbox{-} {\rm{value}} < 0.001,\,{\rm{n}}=44$$

where y = linear length of the colony and x = height of the colony (Supplementary Data S4c).

The AFDM of shallow *E. singularis* was obtained as explained for *P. clavata*, sampling 15 portions of randomly selected colonies (Punta s’Oliguera, Cap de Creus, 42°17′1.62″N, 3°17′57.18″E). For deep populations, 15 pieces of *E. singularis* were randomly collected *in situ* (Es Forcats, Cap de Creus, 42°18′44″N, 3°19′05″E) at a depth of 50–60 m. The AFDM cm^−1^ of shallow *E. singularis* colonies was 5.69 ± 0.47 mg cm^−1^ and for deep colonies it was 4.43 ± 0.37 mg cm^−1^.

The annual increase in height of *E. singularis* was studied by Weinberg & Weinberg^64^ and Munari *et al*.^65^. The mean value of these two studies was used to calculate the C invested in growth by this species. The annual increase in the total length of the colonies was first calculated and subsequently multiplied by the C content of the coenenchyme and axis (mg C cm^−1^) measured by Coma *et al*.^20^ (Supplementary Data S3).

### *Leptogorgia sarmentosa*

Data on the spatial and bathymetric distribution of *Leptogorgia sarmentosa* in the study area are available in Gori *et al*.^15^. As for the other two species, the *L. sarmentosa* observed in the study area were classified as shallow (0–35 m depth) or deep colonies (>35 m depth). Due to the low abundance of this species in the study area, it was not possible to study its population size structure. However, all the colonies encountered were measured as in Gori *et al*.^35^: still images with the presence of *L. sarmentosa* were extracted from the videos, the distance between the laser beams was used as a scale; colonies could be measured only when the laser beams were on the same plane of the gorgonian.

The ingestion rate of live and detrital POM was obtained from previous studies by Rossi^50^ and Ribes *et al*.^23^, whereas the contribution of zooplankton to the diet of *L. sarmentosa* was obtained from Rossi *et al*.^21^. The respiration rates of this species were available only at 16 °C^23^. Although we were aware of the influence of temperature on the respiration process, this was the only data available for this species, and was used in the knowledge that it could introduce some bias into the calculation. The ingestion and respiration rates of *L. sarmentosa* are given as μg C g AFDM^−1^ d^−1^ and mg O_2_ g AFDM^−1^ d^−1^ respectively^23,50^. We first calculated the relationship between colony height (cm) and colony linear length (cm) by means of image analysis: 16 colonies were photographed next to a ruler and the height and length of the colonies were measured, resulting in the following relationship:

$${\rm{y}}=0.21167\,{{\rm{x}}}^{1.3684},\,{\rm{with}}\,{{\rm{R}}}^{2}=0.74,{p} \mbox{-} {\rm{value}} < 0.001,\,{\rm{n}}=16$$

where y = linear length of the colony and x = height of the colony (Supplementary Data S4d).

To calculate the AFDM cm^−1^, 14 pieces of *L. sarmentosa* were randomly collected (Medes Islands, 42°02′55″N, 3°13′30″E) at 25–35 m depth. The AFDM was 2.94 ± 0.67 mg cm^−1^.

Mistri & Ceccherelli^56^ studied the growth rate (height increase) of *L. sarmentosa*. To calculate the C invested in growth by this species, we first calculated the annual increase in total length of the colonies; this value was subsequently multiplied by the C content cm^−1^ obtained by Rossi *et al*.^63^ (Supplementary Data S3).

### Potential role as C sinks of the three gorgonian species

A rough estimate of the potential biomass of the three gorgonians inhabiting the Cap de Creus area was obtained considering the area covered by the suitable benthic assemblages per species^44^ and the density recorded in the 76 transects analysed (Table 3). Based on the potential biomass of the three species in the study area, the relative C flux and C sink have been calculated (Supplementary Data S1a).

The benthic assemblages suitable for *P. clavata* were vertical and platform coralligenous; photophilic algal communities, precoralligenous, vertical and platform coralligenous were suitable for *E. singularis* and photophilic algal communities, vertical and platform coralligenous, littoral sandy mud, medium and coarse sand, detrital littoral sand and sandy mud were suitable for *L. sarmentosa* (Supplementary Data S1b)^44^.

### Statistical analysis

Differences in the biomass, C flux and C sink between the three species were tested by ANOVA and Tukey test, performed with the R software platform^81^. Data were square root transformed to meet the assumption of normality and homogeneity of variance.

## Supplementary information

Supplementary Data S1, S2, S3, S4

## Data Availability

The dataset generated and analysed during the current study is available from the corresponding author on request.
