# Supplementary material for: The importance of coastal gorgonians in the blue carbon budget
Source: Sci Rep. 2019 Sep 19;9:13550. doi: 10.1038/s41598-019-49797-4 (PMC6753119; doi:10.1038/s41598-019-49797-4)
Supplement: Supplementary file 1 — Supplementary Data S1, S2, S3, S4 [file 41598_2019_49797_MOESM1_ESM.pdf]

The importance of coastal gorgonians in the blue carbon budget

Martina Coppari, Chiara Zanella, Sergio Rossi

Supplementary Data S1a Biomass, carbon flux (ingestion – respiration) and sink (growth) of the potential colonies of the three gorgonian species in the Cap de Creus area. Data are standardized to a surface of 116.8 ha, 1068 ha, 9137 ha for *P. clavata*, *E. singularis* and *L. sarmentosa*, respectively[44]

Estimated colonies	Number of colonies	Biomass (kg AFDM)	C flux (kg C d^{-1})	C flux (kg C ha^{-1} d^{-1})	C sink (kg C $year^{-1}$)	C sink (kg C ha^{-1} $year^{-1}$)
Paramuricea clavata	957395	3490.17	16.71	0.25	167.58	2.58
Eunicella singularis	16519606	9727.35	178.42	0.38	2203.78	8.90
Leptogorgia sarmentosa	1488528	412.23	2.50	$7.30*10^{-4}$	76.01	0.02

Supplementary Data S1b Extension of the suitable benthic assemblages per species in the Cap de Creus area[44]. • indicates shallow benthic assemblages; •• indicates deep benthic assemblages

a) *Paramuricea clavata*

**Benthic assemblages**	**Extension (m^2)**
Vertical coralligenous •	365000
Platform coralligenous ••	803000

b) *Eunicella singularis*

**Benthic assemblages**	**Extension (m^2)**
Photophilic algal communities •	6178000
Precoralligenous •	1993000
Vertical coralligenous •	1706000
Platform coralligenous ••	803000

c) *Leptogorgia sarmentosa*

**Benthic assemblages**	**Extension (m^2)**
Photophilic algal communities •	6178000
Precoralligenous •	1993000
Vertical coralligenous •	365000
Platform coralligenous ••	803000
Littoral sandy mud •	44803000
Littoral medium and coarse sand •	10377000
Detrital littoral sands ••	14504000
Detrital littoral sandy mud ••	12347000

Supplementary Data S2

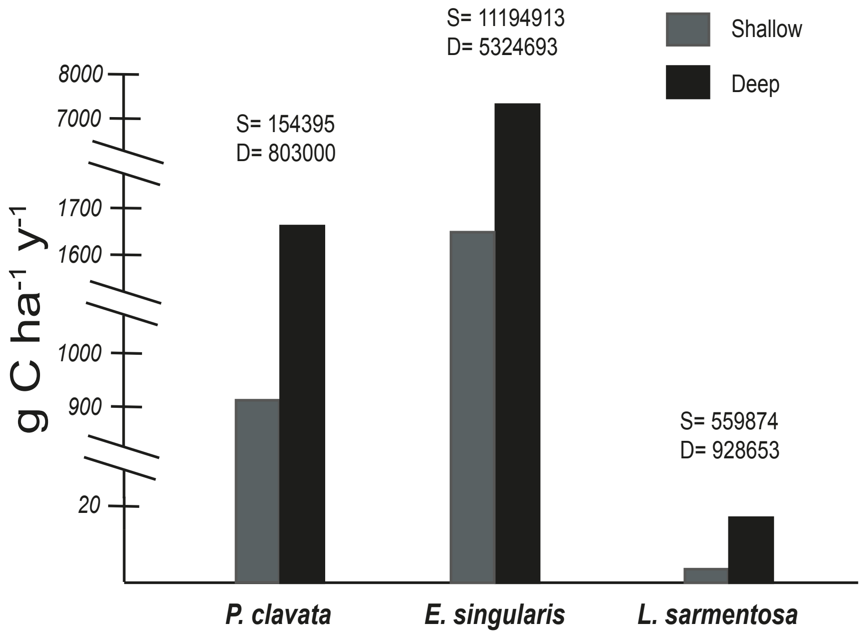

Supplementary Data S3 Previous literature used in this study for *Paramuricea clavata*, *Eunicella singularis* and *Leptogorgia sarmentosa*

Paramuricea clavata	Spatial and bathymetrical distribution	Size distribution	Ingestion	Respiration	Growth
Shallow populations	Gori *et al.*[15]	Linares *et al.*[32]	Coma *et al.*[19]<br>Ribes *et al.*[22]	Previati *et al.*[77]	Coma[79]
Deep populations	Gori *et al.*[15]	This study	Coma *et al.*[19]<br>Ribes *et al.*[22]	Previati *et al.*[77]	Coma[79]

Eunicella singularis	Spatial and bathymetrical distribution	Size distribution	Ingestion	Respiration	Growth
Shallow populations	Gori *et al.*[15]	Linares *et al.*[32]	Coma *et al.*[20]<br>Ribes *et al.*[23]<br>Ferrier-Pagès *et al.*[36]	Previati *et al.*[77]	Weinberg &Weinberg[64]<br>Munari *et al.*[65]
Deep populations	Gori *et al.*[15]	Gori *et al.*[35]<br>This study	Coma *et al.*[20]<br>Ribes *et al.*[23]	Previati *et al.*[77]	Weinberg & Weinberg[64]<br>Munari *et al.*[65]

Leptogorgia sarmentosa	Spatial and bathymetrical distribution	Size distribution	Ingestion	Respiration	Growth
Shallow populations	Gori *et al.*[15]	This study	Rossi *et al.*[21]<br>Ribes *et al.*[23]<br>Rossi[50]	Rossi *et al.*[21]<br>Ribes *et al.*[23]	Mistri & Ceccherelli[56]
Deep populations	Gori *et al.*[15]	This study	Rossi *et al.*[21]<br>Ribes *et al.*[23]<br>Rossi[50]	Rossi *et al.*[21]<br>Ribes *et al.*[23]	Mistri & Ceccherelli[56]

Supplementary Data S4

a)

SHALLOW *Eunicella singularis*

RELATIONSHIP:

HEIGHT-LINEAR LENGTH (cm)

photo code		height (cm)	linear length
DSC03396		32,52	260,14
DSC03398		9,84	22,76
DSC03401		15,21	32,19
DSC0308		8,32	18,64
DSC03410		10,36	7,71
DSC03410		9,88	14,22
DSC03413		7,13	23,90
DSC0314		17,88	62,42
DSC0317		29,54	150,56
DSC0319		27,15	341,15
DSC03421		14,78	79,93
DSC03423		16,72	37,48
DCS03425		11,54	39,19
DCS03427		19,26	129,83
DSC03429		12,68	78,78
DSC03431		15,22	78,64
DCS03434		19,20	71,91
DCS03436		22,41	135,01
DSC03438		12,86	29,16
DSC03440		26,20	140,70
DSC03445		13,82	123,59
DSC03463		35,80	444,66
DSC03455		20,55	117,06
DCS03447		42,00	263,69
DSC03457		33,06	247,63
DSC03467		28,87	383,09
DSC03474		13,65	82,45
DSC03476		13,01	60,76
	**mean**		124,19
	**SD**		118,4693133
	**SE**		22,39
	**n**		28
**95% Confidence Interval for SD**	**Lower**		93,66
	**Upper**		161
**95% Confidence Interval for MEAN**	**Lower**		78,25
	**Upper**		170

**95% Prediction for Observation**	**Lower**	-123
	**Upper**	372

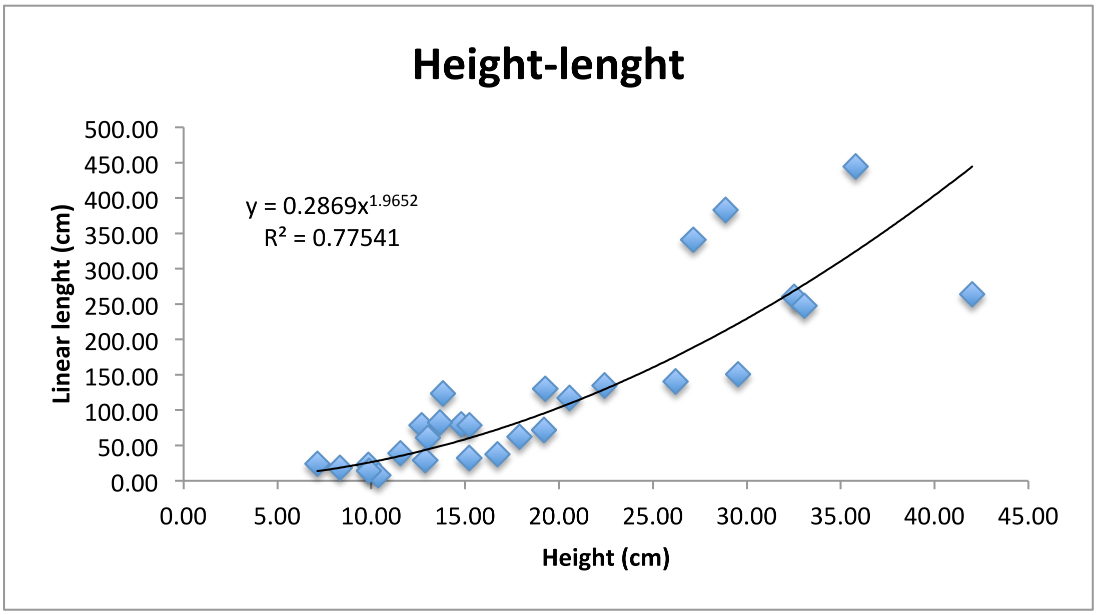

b)

RELATIONSHIP:

HEIGHT-AREA

photo code	height (cm)	area (cm²)
DSC03396	32,52	259,08
DSC03398	9,84	14,30
DSC03401	15,21	27,30
DSC0308	8,32	12,30
DSC03410	10,36	5,33
DSC03410	9,88	11,62
DSC03413	7,13	11,26
DSC0314	17,88	56,87
DSC0317	29,54	165,55
DSC0319	27,15	433,90
DSC03421	14,78	67,03
DSC03423	16,72	30,61
DCS03425	11,54	25,85
DCS03427	19,26	103,33
DSC03429	12,68	64,84
DSC03431	15,22	52,87
DCS03434	19,20	54,97
DCS03436	22,41	109,89
DSC03438	12,86	16,49
DSC03440	26,20	178,01
DSC03445	13,82	104,83
DSC03463	35,80	558,77
DSC03455	20,55	122,79
DCS03447	42,00	341,83
DSC03457	33,06	330,14
DSC03467	28,87	566,16
DSC03474	13,65	74,99
DSC03476	13,01	70,42
	**mean**	138,26
	**SD**	163,1007536
	**SE**	30,82
	**n**	28
**95% Confidence Interval for SD**	**Lower**	129
	**Upper**	222
**95% Confidence Interval for MEAN**	**Lower**	75,02
	**Upper**	202
**95% Prediction for Observation**	**Lower**	-202
	**Upper**	479

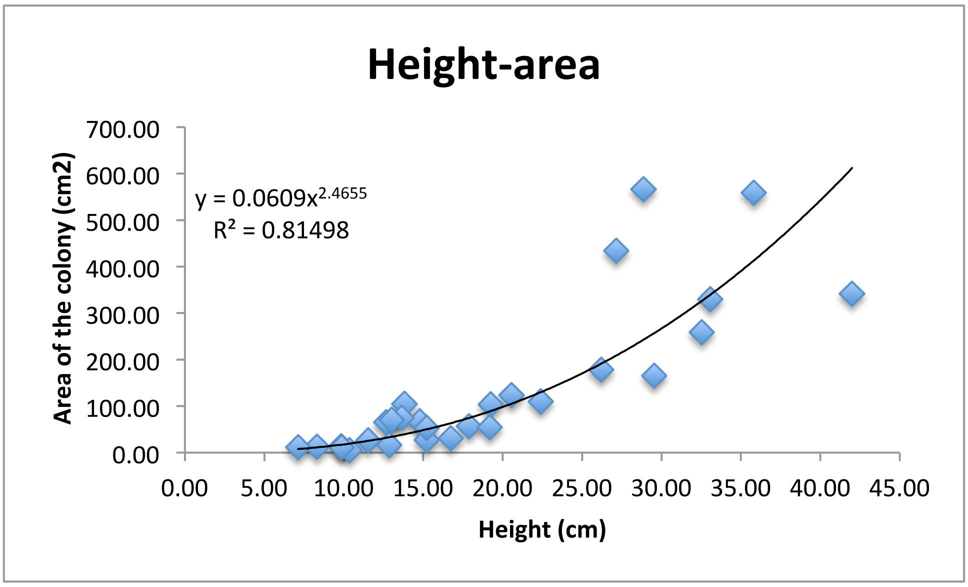

Height-area
y = 0.0609x2.4655
R² = 0.81498
700.00
600.00
500.00
400.00
300.00
200.00
100.00
0.00
Area of the colony (cm2)
0.00
5.00
10.00
15.00
20.00
25.00
30.00
35.00
40.00
45.00
Height (cm)

c)

DEEP *Eunicella singularis*

RELATIONSHIP:

HEIGHT-LINEAR LENGTH (cm)

frame code	height (cm)	linear length (cm)
1	18,16	110,34
2	9,94	26,32
3	22,61	82,53
4	9,04	35,01
5	14,72	28,57
6	11,10	43,83
7	14,24	137,02
8	17,32	106,91
9	9,17	37,92
10	7,18	11,45
11	16,01	110,30
12	8,50	27,28
13	19,15	201,49
14	10,21	103,62
15	9,31	49,78
16	17,13	46,27
17	14,35	53,73
18	18,44	168,03
19	17,38	99,58
20	7,91	9,32
21	12,20	45,00
22	9,56	12,94
23	13,01	39,77
24	8,21	28,05
25	17,02	105,95
26	13,52	55,65
27	15,87	53,58
28	3,20	4,84
29	14,93	58,27
30	7,16	10,03
31	12,46	106,07
32	7,04	117,72
33	12,08	24,61
34	18,13	87,46

35	15,88	48,65
36	4,09	3,95
37	32,84	269,19
38	17,63	104,70
39	8,83	9,75
40	12,89	48,89
41	13,83	127,90
42	7,27	22,04
43	9,13	9,65
44	6,20	15,95
	**mean**	65,91
	**SD**	56,73301746
	**SE**	8,553
	**n**	44
**95% Confidence Interval for SD**	**Lower**	46,87
	**Upper**	71,88
**95% Confidence Interval for MEAN**	**Lower**	48,66
	**Upper**	83,16
**95% Prediction for Observation**	**Lower**	-49,8
	**Upper**	182

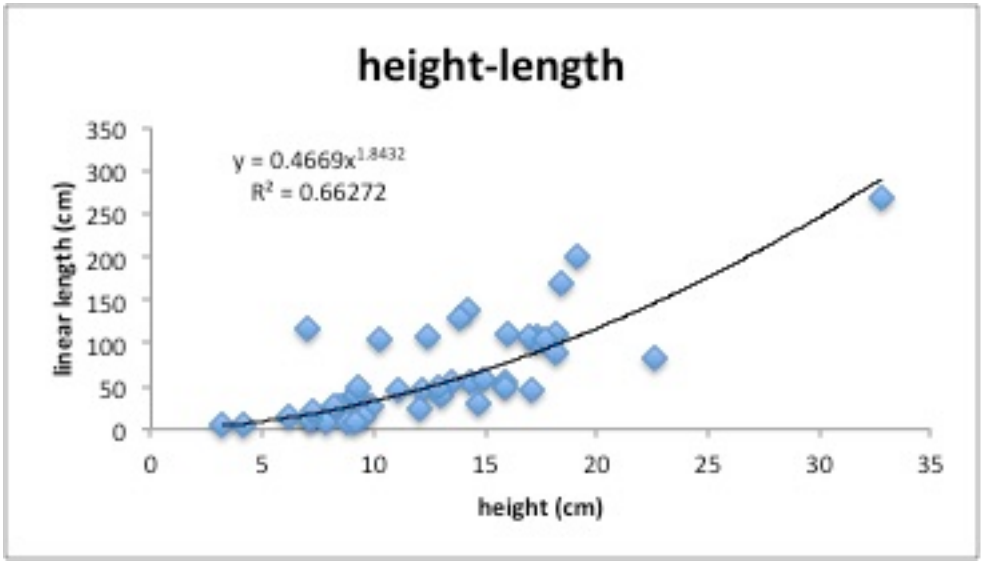

d)

Leptogorgia sarmentosa

RELATIONSHIP:

HEIGHT-LINEAR LENGTH (cm)

photo code		height (cm)	linear length (cm)
**00_01_04_08**		9,30	39,24
**00_01_54_14**		13,92	93,23
**00_02_27_12**		14,33	30,57
**00_03_20_20**		8,84	33,04
**00_04_39_07**		6,8	16,28
**00_05_08_18**		22,92	152,21
**00_05_08_18bis**		26	119,88
**00_09_08_11**		11,82	117,74
**00_06_08_23**		16,13	109,09
**00_09_27_00**		19,85	170,51
**00_10_12_20**		8,54	37,37
**00_10_15_23**		25,12	203,54
**00_12_38_09**		4,86	25,19
**Col_11_30_a**		12,94	92,21
**Col_16_30_a**		8,02	46,15
**Col_5_30_b**		18,36	137,4
	**mean**		88,98
	**SD**		58,42148762
	**SE**		14,61
	**n**		16
**95% Confidence Interval for SD**	**Lower**		43,16
	**Upper**		90,42
**95% Confidence Interval for MEAN**	**Lower**		57,85
	**Upper**		120
**95% Prediction for Observation**	**Lower**		-39,38
	**Upper**		217

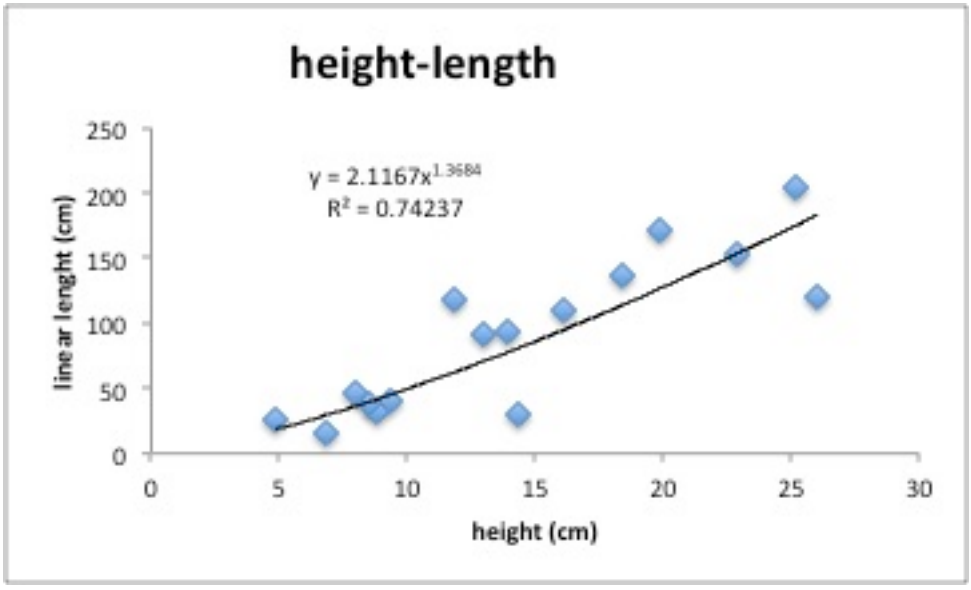

height-length
y = 2.1167x^1.3684
R² = 0.74237
linear lenght (cm)
height (cm)
250
200
150
100
50
0
0
5
10
15
20
25
30
